# Preoperative Body Mass Index, Blood Albumin and Triglycerides Predict Survival for Patients with Gastric Cancer

**DOI:** 10.1371/journal.pone.0157401

**Published:** 2016-06-16

**Authors:** Bin Zheng Liu, Lin Tao, Yun Zhao Chen, Xu Zhe Li, Yu Ling Dong, Ya Jing Ma, Shu Gang Li, Feng Li, Wen Jie Zhang

**Affiliations:** 1 Department of Pathology, the First Affiliated University Hospital, Shihezi University School of Medicine, Shihezi, Xinjiang, China; 2 The Key Laboratories for Xinjiang Endemic and Ethnic Diseases, Shihezi University School of Medicine, Shihezi, Xinjiang, China; 3 Clinical Laboratory, the First Affiliated University Hospital, Shihezi University School of Medicine, Shihezi, Xinjiang, China; 4 Department of Preventive Medicine, Shihezi University School of Medicine, Shihezi, Xinjiang, China; 5 Department of Pathology, Beijing Chaoyang Hospital, Capital Medical University, Beijing, China; University of Nebraska Medical Center, UNITED STATES

## Abstract

**Background:**

Gastric cancer (GC) is common and its prognosis is often poor due to difficulties in early diagnosis and optimal treatment strategies. TNM staging system is useful in predicting prognosis but only possible after surgery. Therefore, it is desirable to investigate prognostic factors/markers that may predict prognosis before surgery by which helps appropriate management decisions preoperatively.

**Methods:**

A total of 320 GC patients were consecutively recruited from 2004 to 2013 and followed up for 127 months (10.6 years) after surgery. These patients’ were examined for body mass index (BMI) and blood levels of albumin, triglyceride, total cholesterol, low density lipoprotein cholesterol (LDL-C), and high density lipoprotein cholesterol (HDL-C). Kaplan-Meier method and log rank test were used to analyze long-term survival using the above potential risk markers. We first employed medians of these variables to reveal maximal potentials of the above prognostic predictors.

**Results:**

Three major findings were obtained: (1) Preoperative BMI was positively correlated with albumin (r = 0.144, *P*<0.05) and triglyceride (r = 0.365, *P*<0.01), but negatively correlated with TNM staging (r = -0.265, P<0.05). Preoperative albumin levels were positively correlated with triglyceride (r = 0.173, *P*<0.05) but again, negatively correlated with TNM staging (r = -0.137, *P*<0.05); (2) Poor survival was observed in GC patients with lower levels of BMI (*P* = 0.028), albumin (*P* = 0.004), and triglyceride (*P* = 0.043), respectively. Receiver operating characteristic (ROC) curve analyses suggested BMI, albumin and triglyceride to have survival-predictor powers similar to TNM system; and (3) Cox multi-factorial analyses demonstrated that age (*P* = 0.049), BMI (*P* = 0.016), cell differentiation (*P* = 0.001), and TNM staging (*P* = 0.011) were independent overall survival-predictors for GC patients.

**Conclusions:**

Preoperative BMI, albumin, and triglyceride levels are capable of predicting survival for GC patients superior to postoperative TNM system in terms of timing for management. As potential survival-predictors, preoperative tests of BMI, albumin and triglyceride, combined with clinical imaging, may help personalized management for GC patients including planning surgical strategy, optimal radio-chemotherapy and appropriate follow-up intervals after surgery.

## Introduction

Gastric cancer (GC) is one of the most common gastrointestinal malignancies in the world posing a great threat to international public health. GLOBOCAN 2012 has reported that GC is the fifth most common cancer (951,600 cases) and the third leading cause of cancer death (723,100 deaths) worldwide [1]. More than 70% of GC cases occur in developing countries, especially in Eastern Asia and China [1]. According to China’s Cancer Statistics, GC is the 2nd most common cancer with 424,000 new cases and 3rd leading cause of death with 298,000 new deaths in 2012 [2]. These above trends remain true in 2015 [3].

If not by screening, early detection of GC is difficult and the management of late stage GC is often unsatisfactory, leading to poor survival. It is consensus that cancer invasion and metastasis are the major causes of death for patients with GC while surgical removal of in situ and metastatic tumors is a routine treatment [4]. However, as TNM clinical staging is not available before surgery [5], there are two dilemmas facing surgery: (1) surgical planning is passive, which may affect the quality of surgery significantly unexpected conditions encountered during surgery; (2) surgical strategies vary dramatically depending on available clinical information and patients’ general fitness[6]. These aforementioned potentials let the prognosis of GC patients be dismal after surgery [7]. Therefore, identifying effective markers before surgery that can predict risk of poor prognosis is very desirable for the following reasons: (1) predicted poor prognosis is a pre-warning that would facilitate active surgical planning and improve the quality of surgery; (2) predicted prognosis would help design appropriate postoperative radio-chemotherapy and optimal intervals of follow-up.

Body mass index (BMI) is a measure of obesity which has recently been indicated to be a risk factor for many common human cancers [8]. On the other hand, studies have suggested that higher BMI is associated with long-term survival in patients with several cancers including GC [9–12], to which we favor the hypothesis that BMI reflects general fitness of GC patients as cancer is a consumptive disease and cachexia is often seen in late and terminal patients with cancer. However, there are also reports suggesting that higher BMI is only an indicator to predict postoperative complications but not associated with overall survival of GC patients [13]. Nevertheless, the role of BMI on the prognosis of GC patients remains controversial [12, 13].

Blood metabolites are also associated with mortality. For example, there are reports suggesting an inverse relationship between blood levels of albumin (Alb) and mortality in the general population [14], in patients with benign [15] and malignant diseases [16–18]. Association studies with patient mortality are also reported, to a lesser extent, in blood levels of lipids, such as triglycerides (TG), total cholesterol (TC), high density lipoprotein cholesterol (HDL-C) and low density lipoprotein cholesterol (LDL-C). A cohort study has reported that abnormal TG levels increase the risk of lung cancer and rectal cancer [19]. Another study, however, has shown that TG levels are not associated with the risk of cancer mortality [20].

This study has investigated potentials of less well known risk factors that impact on survival including those obtained preoperatively such as BMI and blood metabolites, and those obtained postoperatively such as TNM staging system in a GC patient cohort with the longest follow-up period of 127 months. Three major findings have been obtained: (1) BMI, Alb, and TG are positively correlated with each other, however, negatively correlated with at least one of the TNM staging factors; (2) GC Patients with lower levels of BMI, Alb or TG have poorer survival prognosis; and (3) BMI is an independent factor capable of predicting survival among GC patients.

## Materials and Methods

### Ethics Statement

This study was approved by the Institutional Ethics Review Board (IERB No. SHZ2010LL03) at our First Affiliated Hospital, Shihezi University School of Medicine. The IERB waived the need of patients’ consent due to anonymous analyses of the data and standard university hospital guidelines in accordance with the Declaration of Helsinki including confidentiality and anonymity were followed in the handling and publication of patients’ tissues.

### Patients and Follow-Up

Information from 320 consecutive GC patients including 237 males and 83 females undergone surgery was obtained from January 1, 2004 to June 30, 2013 from the First Affiliated Hospital, Shihezi University School of Medicine. GC diagnoses were re-confirmed by a senior pathologist. Survival time was considered from the date of surgery until the date of death or the date of the last follow-up. Follow-up ranged from 0 to 127 months (10 years and 7 months) after surgery until October 2, 2014 and the median follow-up time was 30 months. There were 10 patients who died within 30 days after surgery. The patients were aged from 27 to 86 years (median 64 years). By October 2, 2014, 169 (52.8%) patients died and 151 (47.2%) GC patients were still alive. Patients who died within 30 days after surgery were recorded as 0 month follow-up or survival. Patients’ demographic and clinicopathological characteristics were summarized in Table 1.

**Table 1:** Clinicopathological characteristics, ranges, reference ranges and median ranges of age, BMI, blood albumin and lipids among 320 GC patients.

Characteristics	Patients (n = 320)		Characteristics (cont'd)	Patients (n = 320)	
	n	%		n	%
**Sex**			**BMI (kg/m**^**2**^**)**		
Male	237	74.1	Range	15.1–32.2	
Female	83	25.9	Median	22.7	
**Age (years)**			UW (15.1–18.5)	24	10.4
Range	27–86		NW (18.6–23.9)	131	56.7
Median	64		OW (24.0–27.9)	56	24.2
Mean	60		OB (28.0–32.2)	20	8.7
<60	120	37.5	**Blood albumin (g/L)**		
>/ = 60	200	62.5	Range	20.3–49.7	
**Cell differentiation**			Median	39.1	
Well	14	4.4	Normal range	40–55	
Moderate	94	29.4	20.3–39.9	136	44.2
Poorly	212	66.2	40.0–49.7	172	55.8
**Follow-up (months)**			**Triglyceride (mmol/L)**		
Range	0–127		Range	0.1–7.58	
Median	30		Median	0.96	
Mean	42		Normal range	<1.7	
**Invasion depth**			0.1–1.6	133	88.1
T1	36	11.3	1.7–7.58	18	11.9
T2	64	20	**Total cholesterol (mmol/L)**		
T3	212	66.2	Range	2.01–6.43	
T4	8	2.5	Median	4.0	
**Lymph node metastasis**			Normal range	<5.18	
No	134	41.9	2.01–5.17	132	87.4
Yes	186	58.1	5.18–6.41	19	12.6
**Distant metastasis**			**LDL-C (mmol/L)**		
No	290	90.6	Range	0.81–4.76	
Yes	30	9.4	Median	2.36	
**TNM staging**			Normal range	<3.37	
I	72	22.5	0.1–3.36	127	84.1
II	110	34.4	3.37–4.76	24	15.9
III	107	33.4	**HDL-C (mmol/L)**		
IV	31	9.7	Range	0.39–2.96	
			Median	1.12	
			Normal range	<1.04	
			0.39–1.03	64	42.4
			1.04–2.96	87	57.6

Note: Patients who died within 30 days after surgery were defined as 0 month of follow-up or survival; UW = underweight; NW = normal weight; OW = overweight; OB = obesity; LDL-C = low-density lipoprotein cholesterol; HDL-C = high-density lipoprotein cholesterol.

### Measurements of BMI, Blood Metabolites, and TNM Staging

Patients received no chemotherapy and/or radiotherapy before surgery when they were measured for BMI and blood metabolites (bio-indicators). Body weight and height were measured with light clothing and no shoes and BMI was calculated as weight (kilogram or kg) divided by height (meter or m) squared (kg/m^2^). According to the Chinese Working Group on Obesity [21–23], underweight was defined as BMI</ = 18.5 kg/m^2^, normal weight as 18.6 to 23.9 kg/m^2^, overweight as BMI>/ = 24 to 27.9 kg/m^2^, and obesity as BMI>/ = 28 kg/m2, respectively. The Median BMI was 22.7 kg/m^2^ in this cohort (Table 1).

Blood samples (3 ml) were collected from patients in the morning following a 12-hour overnight fasting. Blood was drawn into a tube, centrifuged at 2500–3000 rpm for 10–15min, and serum was stored at 4°C until biochemical analyses on the same day. Blood metabolites were measured in Clinical Biochemistry Laboratories at the First Affiliated Hospital, Shihezi University School of Medicine. Serum albumin (Alb) and lipids were measured using a Roche Modular D/P automated analyzer (Roche Modular Analytics and reagent kits supplied by Roche Company, USA). Alb and triglyceride (TG) were measured using bromcresol green method. Total cholesterol (TC) and high density lipoprotein cholesterol (HDL-C) were analyzed by enzymatic method and low density lipoprotein cholesterol (LDL-C) was analyzed by catalase assay. Normal reference ranges for the above blood metabolites (Table 1) were according to Chinese National Guide to Clinical Laboratory Test Procedures [24]. Tumor TNM staging was determined based on surgical findings according to the AJCC TNM staging system (2010) [5] (Table 1).

### Statistical Analyses

**Fig 1:**
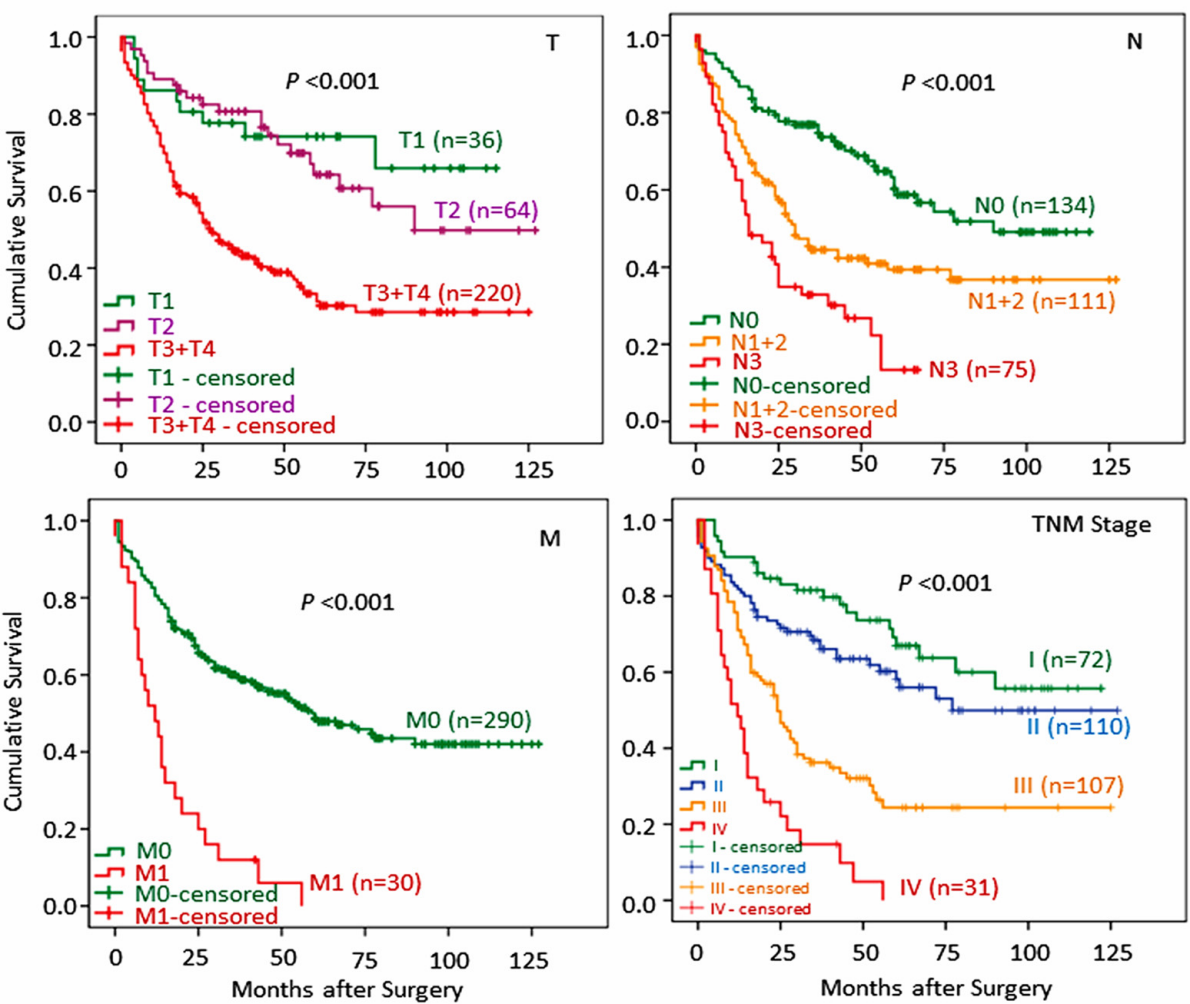
Survival analyses against the established risk factors affecting survival validate the GC patient cohort for analyzing potential new factors. The cohort included 320 GC patients, of whom 151 were alive and 169 were dead. To validate the cohort for survival analysis, general survival is analyzed against several established factors affecting survival in GC patients using Kaplan-Meier method. Panel T shows the impact of tumor infiltration depth (T1, T2, T3 and T4) on the survival with the T3 and T4 patients showing the poorest survival. Panel N demonstrates that the more metastatic lymph nodes (from N0 to N3) in a patient, the poorer the survival. Panel M indicates that patients with distant metastasis (M1) have worse survival than patients without metastasis (M0). Panel TNM staging depicts a typical survival hierarchy that patients with the earliest stage I have the best survival while patients with the latest stage IV show the poorest survival. T, N, M, and TNM staging were determined according to the AJCC TNM staging system [5].

**Fig 2:**
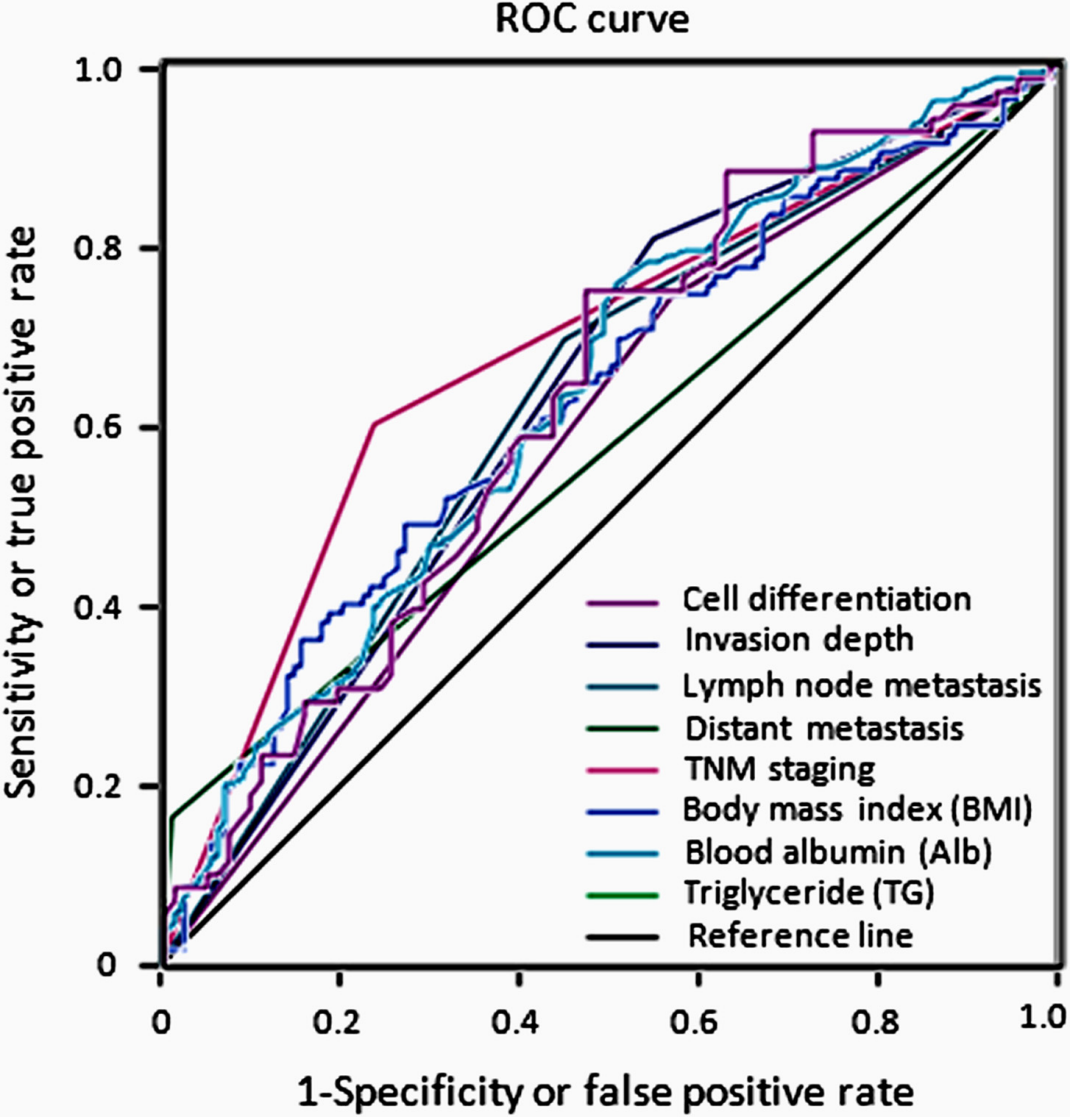
ROC curves reveal performance abilities for 8 factors affecting patient survival. As shown, the diagonal black line is the reference line and, TNM staging has the largest AUC (purple line, AUC = 0.685) followed by BMI (blue line, AUC = 0.636), Alb (light blue line, AUC = 0.633), and TG (dark green line, AUC = 0.629) (see Table 4 for statistical comparisons). It is very interesting to note that comparisons indicate the 3 new potential survival predictors TG, Alb, and BMI to have AUCs similar to, or even better than, AUCs of the conventional survival predictors T, N, M, and TNM staging (see Table 4, list of individual AUCs). In keeping with Figs 1, 3, 4 and 5, these AUC observations again demonstrate that these newer predictors have very similar, if not the same, power in predicting survival prognosis as do the conventional survival predictors.

Statistical analyses were performed using the SPSS statistical software package (version 17.0). Spearman’s rank correlation method was used to analyze correlations between bio-indicators. Survival curves were drawn by Kaplan-Meier method and differences were assessed by Log-rank test. However, Kaplan-Meier analysis only classifies and compares one variable and cannot make comparisons among different variables to judge abilities among variables as they may be all capable of predicting survival (see Fig 1). Therefore, potential survival predictors have to be quantified so they can be compared. For this purpose, we introduced the receiver operating characteristic (ROC) curve and the area under the curve (AUC) to quantify powers or abilities of survival-predictors. ROC is a graphical plot that illustrates the performance of a binary classifier system as its discrimination threshold is varied [25]. AUC is a product of considering both sensitivity and specificity (Fig 2) which can be translated to discriminative power. Therefore, the larger the AUC is, the more powerful the risk factor can be in predicting prognosis. Scatter diagram method was used to evaluate correlations of BMI, Alb, and TG with overall survival time among patients with GC.

To test if blood metabolites could have true impacts on survival, we used two different grouping systems in the analyses: (1) patients were grouped based on normal reference ranges, which is often used by most, if not all, published survival analyses; (2) we first introduced a grouping of patients based on median figures (see Table 1) and this grouping would have more power to show potential differences in survival between two terminal ends of a variable as blood metabolites could be extremely imbalanced in cancer patients. Cox proportional hazards regression model was conducted to evaluate the independence of variables that impact on prognosis. *P* values of <0.05 were considered significant statistically.

## Results

### Correlations among Blood Metabolites and TNM Staging System

We hypothesized that, if BMI and blood metabolites had impacts on prognosis, they would show correlations with conventional TNM-defined system. We therefore first tested correlations among BMI, blood metabolites and TNM staging. As shown in Table 2, it was interesting to note that BMI was positively correlated with Alb (r = 0.144, *P*<0.05), TG (r = 0.365, *P*<0.01), LDL-C (r = 0.233, *P*<0.05) but negatively correlated with T (r = -0.342, P<0.01), N (r = -0.208, P<0.05), TNM staging (r = -0.265, *P*<0.05), respectively. Protein Alb was found to be correlated with TNM staging (r = -0.137, *P*<0.05), TG (r = 0.173, *P*<0.05), and LDL-C (r = 0.310, *P*<0.001) respectively. In addition, TG was negatively correlated with T (r = 0.203, *P*<0.05).

**Table 2:** Correlations among preoperative BMI, Alb, blood lipids and clinicopathological features in gastric cancer patients.

Variables(n)	Cell diff. (320)	T (320)	N (320)	M (320)	TNM (320)	BMI (231)	Alb (308)	TC (151)	TG (151)	HDL-C (151)	LDL-C (151)
Cell diff.	1										
T	0.144	1									
N	0.232*	0.368**	1								
M	0.039	0.097	0.111	1							
TNM	0.227*	0.684***	0.761**	0.464**	1						
BMI	-0.108	-0.342**	-0.208*	-0.147	-0.265*	1					
Alb	0.061	-0.112	-0.082	-0.074	-0.137*	0.144*	1				
TC	-0.004	-0.03	0.053	-0.179*	-0.056	0.181	0.061	1			
TG	-0.102	-0.203*	-0.127	-0.041	-0.127	0.365**	0.173*	0.358**	1		
HDL-C	-0.067	0.018	0.030	0.065	0.024	-0.243*	0.064	0.253*	-0.39**	1	
LDL-C	-0.073	-0.082	0.046	-0.101	-0.024	0.233*	0.310***	0.779**	0.391**	0.036	1

Note: Values in the main table are correlation coefficient or *r* values. The variables below the short middle line are obtainable before surgery. (n) represents the number of patients with that variable tested/available. Cell diff. = cell differentiation; T = invasion depth; N = lymph node metastasis; M = distant metastasis; TNM = TNM staging; BMI = body mass index; Alb = albumin; TC = total cholesterol; TG = triglyceride; HDL-C = high-density lipoprotein cholesterol; LDL-C = low-density lipoprotein cholesterol

* = *P*<0.05

** = *P*<0.01

*** = *P*<0.001. Two representative analyses using scatter diagram method are displayed in S1 Fig.

### TNM-Defined Late Stage GC Patients Show Poor Prognosis

To test whether new or less known markers, such as blood metabolites, play a role in survival prognosis, this GC patient cohort needs to be validated first. We accordingly analyzed general survival of these GC patients against several established conventional risk factors that affected survival using Kaplan-Meier method and Log-rank test. The cohort included 320 GC patients with 151 alive and 169 dead at the last follow-up. As shown in Fig 1, it was immediately obvious that TNM system, including tumor infiltration depth (T), lymph node metastasis (N), distant metastasis (M) and TNM staging, nicely correlated with general survival among these GC patients in a clear dose/severity-dependent manner. Having validated the patient cohort in terms of conventional risk factors for survival, we became confident and went to analyze those less known risk factors for survival as described below.

### BMI Correlates with Survival Prognosis in GC Patients

**Fig 3:**
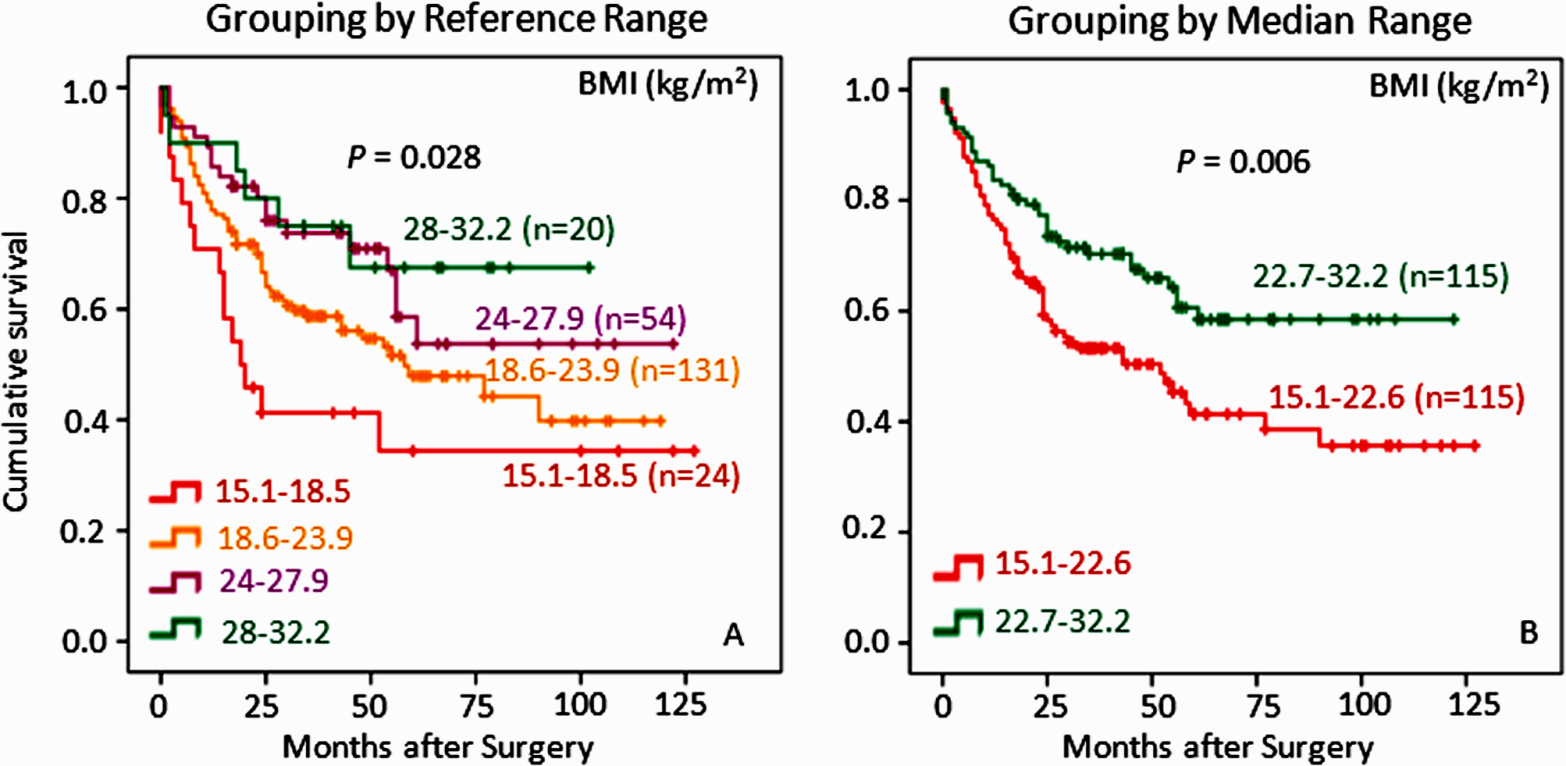
Low levels of BMI correlate with poor overall survival for GC patients by both reference range grouping and median range grouping. Kaplan-Meier survival graphs are shown using either clinical reference ranges (left panel) or median ranges (right panel). It is clear that poorer survival is seen in patients with lower BMI by either reference range or median range. It is also interesting to note that grouping by median range appears to have more statistical power in terms of probability or *P* value to predict potential differences in survival rates among GC patients than grouping by reference range. The censored patients in Kaplan-Meier graphs are represented by a dot in the line.

As shown in Fig 3, Kaplan-Meier survival curves indicated that BMI, when grouped by reference range (Fig 3A), correlated well with survival in a dose-dependent manner, i.e., the lower the BMI, the worse the survival and, vice versa. For example, patients with BMI 24–32.2kg/m^2^ (defined as overweight and obese) exhibited better survival than patients with BMI 15.1–24kg/m^2^ (defined as normal and underweight). Indeed, the trend of BMI’s impact on survival remained true when patients were grouped in median range (Fig 3B), suggesting that lower BMI is likely a real risk factor for GC survival in keeping with the observational analysis that BMI is an independent factor (Table 3). Furthermore, it should be emphasized that BMI was negatively correlated with general TNM system (Table 2) suggesting that BMI may be useful in predicting not only survival but also early stage of disease in GC patients.

**Table 3:** Cox regression analyses indicate that preoperative variables of age, BMI and cell differentiation and, postoperative variable of TNM stage are independent risk factors for survival prognosis in GC patients.

Variables	Univariate Analysis			Multivariate Analysis		
	HR	95% CI	*P* value	HR	95% CI	*P* value
Age (>/ = 60 *vs* <60)	1.427	(1.031–1.975)	0.032	1.958	(1.003–3.820)	0.049
Sex (Male *vs* Female)	1.090	(0.773–1.537)	0.625	/	/	/
Albumin (<40 *vs* >/ = 40 mmol/L)	0.632	(0.459–0.870)	0.005	/	/	/
TG (>0.96 *vs* <0.96 mmol/L)	0.613	(0.378–0.994)	0.047	/	/	/
TC (>/ = 5.18 *vs* <5.18 mmol/L)	0.694	(0.300–1.606)	0.393	/	/	/
HDL-C (>/ = 1.04 *vs* <1.04 mmol/L)	0.849	(0.457–1.578)	0.605	/	/	/
LDL-C (>/ = 3.37 *vs* <3.37 mmol/L)	0.700	(0.367–1.338)	0.28	/	/	/
BMI (UW+NW *vs* OW+OB)	0.574	(0.365–0.903)	0.016	0.314	(0.122–0.804)	0.016
Cell diff. (Poor *vs* Moderate+Well)	1.869	(1.321–2.643)	<0.001	3.862	(1.659–8.803)	0.001
Invasion depth (T3+4 *vs* T1+2)	2.762	(1.875–4.070)	<0.001	/	/	/
Lymph node metastasis (Yes *vs* No)	2.210	(1.589–3.073)	<0.001	/	/	/
Distant metastases (Yes *vs* No)	3.585	(2.367–5.430)	<0.001	/	/	/
TNM stage (III+IV *vs* I+II)	3.110	(2.270–4.261)	<0.001	2.333	(1.219–4.466)	0.011

Note: / = in Multivariate Analysis, results with *P* >0.05 are not displayed. HR = hazard ratio; CI = confidence interval; Cell diff. = cell differentiation by WHO histological type. UW = underweight; NW = normal weight; OW = overweight; OB = obesity; TC = total cholesterol; TG = triglyceride; HDL-C = high-density lipoprotein cholesterol; LDL-C = low-density lipoprotein cholesterol. Variables above the middle line (above Invasion depth) are tested/obtained before surgery.

### Albumin and Triglyceride Positively Correlate with Survival in GC Patients

**Fig 4:**
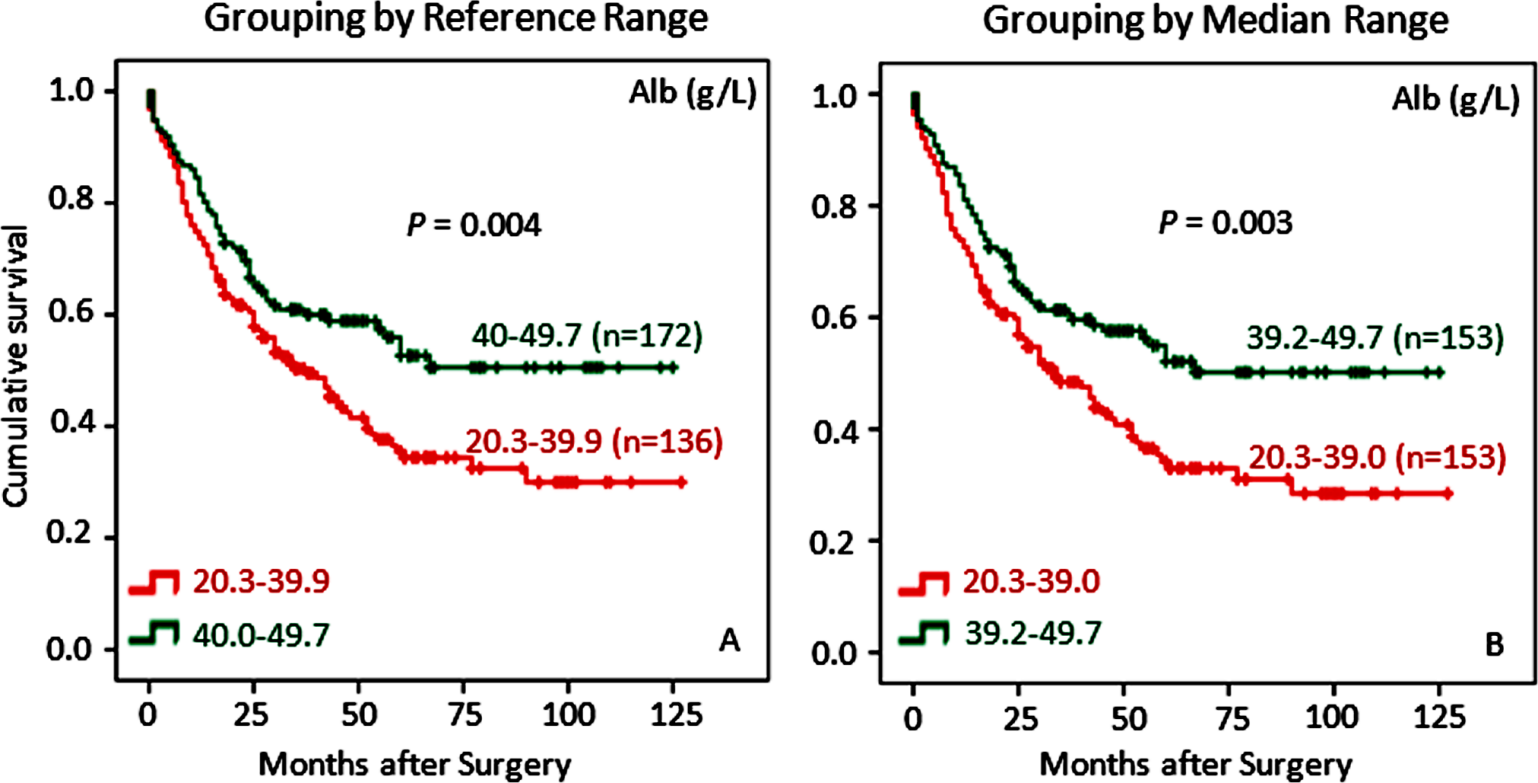
Low levels of albumin (Alb) correlate with poor overall survival for GC patients by both reference range grouping and median range grouping. Similar to BMI analyses as shown in Fig 3, GC patients with lower levels of Alb have worse survival according to either clinical reference range (left) or median range (right) than those with higher levels of Alb. Again, grouping by median range appears to have more statistical power in terms of probability (*P* value) to predict potential differences in survival rates among GC patients. The censored patients in Kaplan-Meier graphs are represented by a dot in the line.

Cancer is a wasting disease and GC is particularly so because of damaged digestive function, so late stage patients with GC often deteriorate to cachexia along with disease progression. Alb can be used to assess the nutritional status in patients and, in the case for GC patients, Alb is useful in assessing cachexia or the progression of disease prior to surgery. Indeed as shown in Fig 4A, GC patients with higher levels of Alb (40–49.7 g/L) showed better survival than those with lower levels of Alb (20.3–39.9 g/L), and this was also true when patients were categorized according to the median range (Alb 39.2–49.7 g/L vs. 20.3–39.0 g/L, Fig 4B).

**Fig 5:**
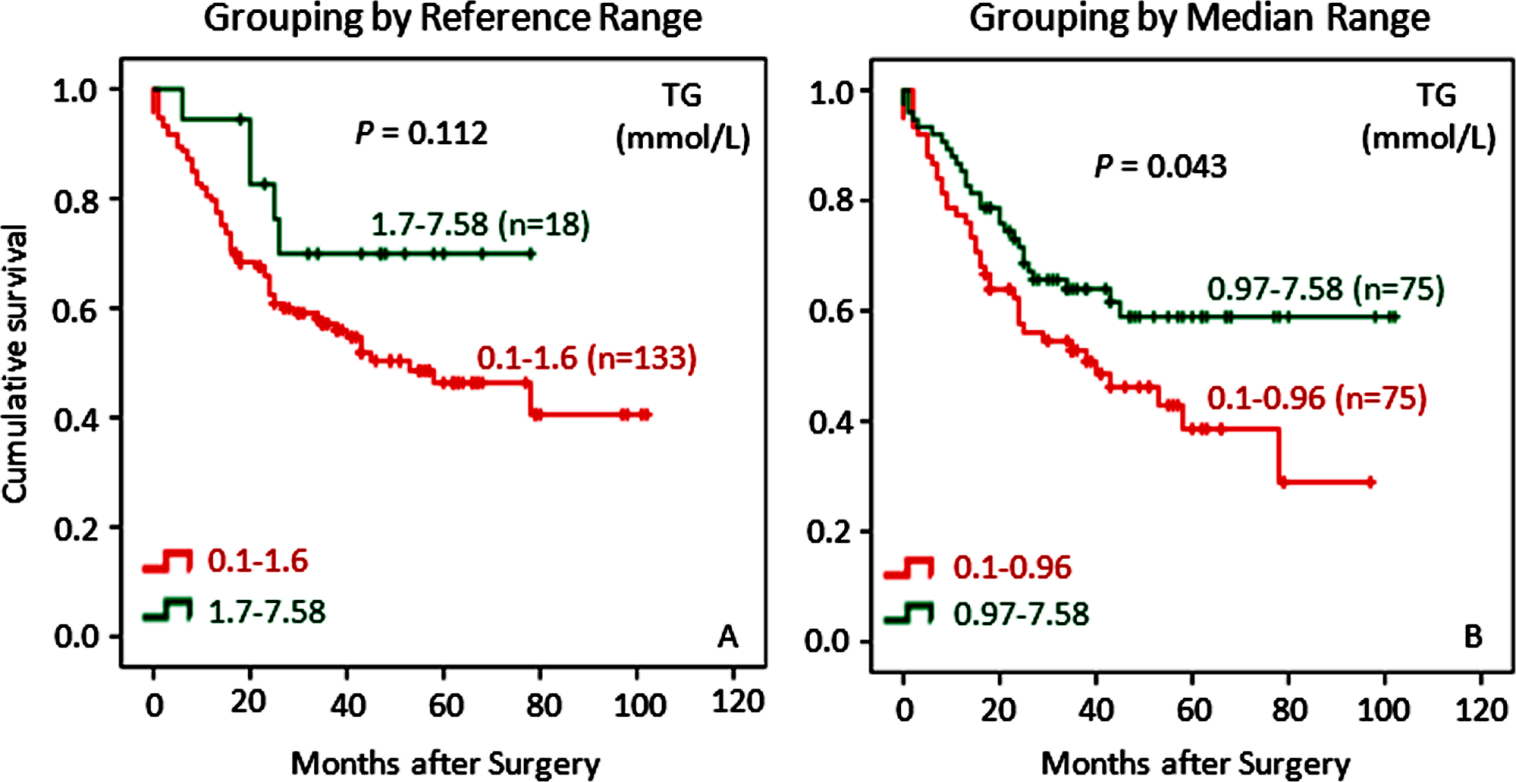
Low levels of TG correlate with poor overall survival for GC patients by both reference range grouping and median range grouping. Triglyceride (TG) shows a trend that, although not statistically significant (*P* = 0.112), patients with lower TG levels have inferior survival to patients with higher TG levels when grouped by reference range (left panel); however, this trend becomes significant (*P* = 0.043) when patients are grouped by median range (right panel). Again, it is evident that grouping by median range shows more statistical power in terms of probability or *P* value to predict potential differences in survival rates among GC patients. The censored patients in Kaplan-Meier graphs are represented by a dot in the line.

Blood lipids are stored form of energy and participate in many important functions. As can be seen in Fig 5A, patients with higher levels of triglyceride (TG) were better off in survival versus those with lower TG levels although not statistically significant (*P* = 0.112). The grouping by median range of TG levels, again, appeared to be able to discriminate better survival in patients with higher TG levels than those with lower TG levels (P = 0.043) (Fig 5B). To the best of our knowledge, this study was the first to introduce median grouping in Kaplan-Meier survival analysis. Thus far, our analyses failed to show the impacts on survival by total cholesterol (TC), high-density lipoprotein cholesterol (HDL-C) and low-density lipoprotein cholesterol (LDL-C), either by reference range or median range (data not shown).

To investigate if surgery- or other condition-related deaths would affect the general trend of survival, we performed survival analyses by removing the 10 patients who died within 30 days after surgery, by which the survival trends remained unchanged for BMI, Alb and TG, respectively (S2 Fig), as compared with survival analyses of all cases inclusive (Figs 3, 4 and 5).

**Fig 6:**
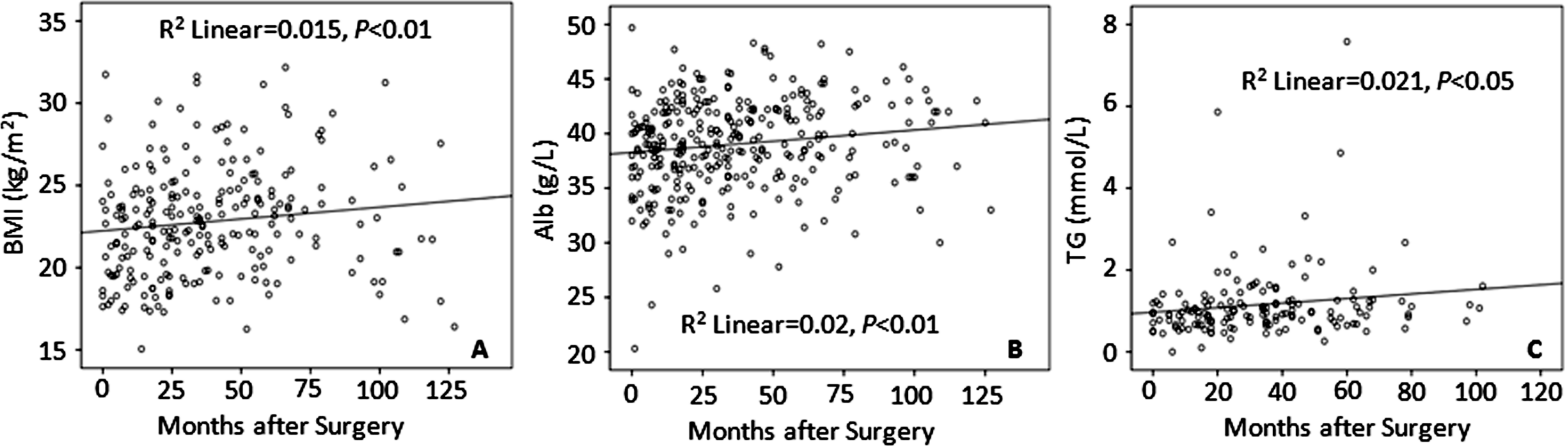
BMI, Alb and TG are positively correlated with overall survival time among patients with gastric cancer. As shown, the positive correlations of these 3 prognostic markers with overall survival time of gastric cancer patients are revealed using scatter diagram method, in keeping with the survival curves analyzed using Kaplan-Meier method (see Figs 3, 4 and 5).

On the other hand, we also analyzed the prognostic values of BMI, Alb and TG using scatter diagram method and found that these three markers were positively correlated, respectively, with overall survival time (month) among these patients with gastric cancer (Fig 6), consistent with the survival curves analyzed by Kaplan-Meier method (Figs 3, 4 and 5).

### BMI and Age Are Independent Factors Affecting GC Patients’ Survival

Among 13 individual factors investigated here, there were 9 factors that affected survival in this GC patient cohort (Table 3). We then asked which ones of these risk factors would act independently on GC patient survival. As seen in Table 3, comprehensive TNM staging, but not individual TNM components that were used to define TNM staging, acted independently on GC patient survival, in keeping with those established observations [26]. It was interesting to note that age (HR = 1.958, *P* = 0.049), cell differentiation (HR = 3.862, *P* = 0.001), and BMI (HR = 0.314, *P* = 0.016) appeared to be independent factors, of which BMI was shown to be a protective factor affecting survival (Table 3). More importantly, these 3 factors, i.e., age, BMI, and cell differentiation are obtainable before surgery suggesting that GC patients’ prognoses may be predicted that would help decide appropriately surgical strategies preoperatively.

### Quantified Predicting Powers of Survival Predictors Using ROC Curves

Kaplan-Meier analysis compares groupings within one variable for its power (ability) to discriminate differences in survival, but cannot make comparisons among different survival variables because they are not quantified. We first used ROC curve and AUC (area under the curve) to quantify the power of a survival predictor in patients with gastric cancer, by which compares the potentials of different survival predictors. As described in Methods, the larger the AUC is, the more powerful the risk factor can be in predicting prognosis. As shown in Table 4 and Fig 2, TNM staging had the largest AUC of 0.685. However, it was somewhat surprising that 3 preoperatively obtained predictors, BMI, Alb, and TG showed similar AUCs (0.636, 0.633, and 0.629, respectively) to those of postoperatively obtained T, N, and M (AUC = 0.629, 0.625, and 0.576, respectively), the 3 components used to define TNM staging (Table 1). Similar AUCs suggest similar discriminative powers in predicting prognosis, which indicates the clinical usefulness of BMI, Alb, and TG in predicting survival in GC patients (Figs 3, 4 and 5).

**Table 4:** Preoperative survival predictors BMI, ALB and TG have similar AUCs or predicting powers (abilities) to the established TNM system in predicting survival in GC patients.

Survival Predictors	AUC	95% CI	*P* value
Reference curve	0.500	N/A	N/A
BMI	0.636	0.563–0.708	<0.001
Alb	0.633	0.572–0.695	<0.001
TG	0.629	0.541–0.717	0.006
Cell differentiation	0.580	0.517–0.643	0.013
T	0.629	0.567–0.691	<0.001
N	0.625	0.564–0.687	<0.001
M	0.576	0.514–0.638	0.018
TNM	0.685	0.626–0.743	<0.001

Note: AUC denotes area under the curve (see Fig 2). 95% CI = 95% confidence interval; N/A = not applicable; T = invasion depth; N = lymph node metastasis, M = distant metastasis; TNM = TNM staging; BMI = body mass index; Alb = albumin; TG = triglyceride. *P* values are the products derived from the comparisons between individual AUCs of the survival predictors, respectively with the reference AUC of 0.500 (see black diagonal line in Fig 2). The 4 predictors above the middle line are obtained before surgery and the 4 predictors under the middle line are obtained after surgery.

## Discussion

In general, the 5-year survival rate for GC patients is low worldwide and this rate is only 31.3% in China, far lower than that in South Korea (58%) and in Japan (54%) [27]. This low survival rate is indicative of late diagnosis and inadequate management, including surgery and radio-chemotherapy. Early diagnosis of GC can be achieved through awareness education and screening and, appropriate management is a complex task involving many considerations. Like all other cancers, GC patient’s survival after surgery is a complex function determined by comprehensive interactions of multiple factors, known and unknown, but a well-designed surgical strategy is critical for better prognosis due to complexities of individual GC cases [6].

Conventional TNM staging system [26], based on tumor invasion (T), lymph node metastasis (N) and distant metastasis (M), has been established to be able to predict survival of GC patients. We have used TNM staging system to validate our GC patient cohort before testing new or less established prognostic factors. As seen in Fig 1, T, N, M, and TNM staging all have shown impacts on survival as expected. TNM staging system, however, is only possible after surgical findings of T, N, and M, making it little reference value for planning an appropriate surgical strategy. It appears desirable to define preoperative bio-markers that have (1) relationships with TNM staging system, and (2) abilities to predict survival prognosis for GC patients. In this study, we have tested several preoperatively obtained bio-markers, such as BMI and blood metabolites, and analyzed their relationships with conventional TNM staging and abilities to predict survival prognosis preoperatively for GC patients.

We have hypothesized that, if BMI and blood metabolites have impacts on GC prognosis, they should show relationships with conventional TNM-defined system which is an established survival predictor. Indeed, we have observed two categories of correlations. First category is positive correlations among preoperative BMI and blood metabolites Alb and TG (Table 2) and second category is negative correlations of BMI, Alb and TG with conventional TNM system (T, N, M and TNM staging, respectively) (Table 2). The second category of correlations is interesting as such correlations suggest some intrinsic relationships between TNM system and BMI, Alb and TG. In other words, BMI and certain metabolites may potentially imply cancer progression as defined by TNM system. These relationships further imply that BMI and metabolites may be able to predict prognosis just as TNM system does for GC patients (Fig 1). Indeed, our observations demonstrate that BMI, Alb and TG have exhibited such prediction capabilities in the survival of GC patients.

BMI is easily measured and used to diagnose overweight and obesity [28]. The relationship between underweight and worse outcomes in GC patients has been reported [12, 29]. However, this unexpected association is referred to as the “obesity paradox” hypothesis [30–32]. The hypothesis describes the paradoxically better outcomes of overweight and obese patients, which is in contrast to the usually held belief that high BMI is a risk factor of death in the general population [29, 33, 34]. Our results show three important observations. First, GC patients with higher BMI have better survival (Fig 3). Second, BMI appears to be an independent protective factor for GC patients in terms of survival (Table 3). Third, BMI is negatively correlated with TNM system components (Table 2). Based on these findings, we favor the hypothesis that overweight and obese individuals represent a category of GC patients who are diagnosed early and less progressive relative to patients with lower BMI. Because GC is a malignant digestive disease often with severe weight loss and cachexia, patients with higher BMI may be physically better off in terms of malnutrition [35]. Our view is supported by the evidence that BMI is positively correlated with blood Alb and lipids (Table 2). Further evidence is when GC patients lose their weight above 10% before surgery, they will have poorer prognosis than those who lose less weight [36]. In the case of already diagnosed GC patients, higher BMI, together with associated blood Alb and lipids (Table 2), may be more relevant as an indication of nutritional status than as a risk factor of GC.

As one of the major blood proteins indicative of individuals’ nutritional status, low levels of Alb have been reported to be an independent prognostic factor negatively associated with survival in several cancers including gastric cancer, lung cancer, colorectal cancer, and breast cancer [37–39]. Our study has shown that Alb levels of 20.3–39.9 g/L (reference range) is predictive of poor prognosis for GC patients (Fig 4), in keeping with the previous observations as mentioned above. Furthermore, additional analysis using median numbers demonstrates that Alb levels below the median predicts poor survival for GC patients (Fig 4), supporting the above finding by reference range analysis. Accordingly, it makes sense that Alb levels are correlated with BMI categories and both are negatively associated with TNM stages (Table 2). These observations support the above hypothesis that BMI and its associated nutritional status suggest a subcategory of GC patients who are diagnosed early, which may explain their better survival prognosis.

The relationship between TG and cancer morbidity and/or mortality is unclear. Association studies between triglyceride and colorectal cancer have shown negative findings [20]. Using an adjusted model, another study has suggested TG to be a risk factor for female GC patients [40]. However, one investigation suggests that TG concentrations are inversely associated with non-Hodgkin’s lymphoma and prostate cancer but positively with lung and rectal cancers [19]. To the best of our knowledge, there are no previous studies exploring the relationship between TG and survival prognosis in patients with only GC. We have observed a phenomenon that lower levels of TG are associated with poorer survival in GC patients than higher levels of TG using median analysis (Fig 5). TG is one of the major lipid metabolites involved in energy supply, and there would be no surprise that blood TG is correlated with both BMI and blood Alb (Table 2). These findings, again, support the hypothesis that higher BMI and its associated nutritional status suggest the presence of a subcategory of GC patients with early diagnosis and less progression.

Furthermore, it should be noted that, in terms of the prognostic values of BMI, Alb and TG, we have observed similar results that BMI, Alb and TG are positively correlated with overall survival time (month) among these patients with gastric cancer using scatter diagram method (Fig 6), in keeping with the survival curves analyzed using Kaplan-Meier method (Figs 3, 4 and 5).

Kaplan-Meier analysis compares groupings within one variable for its power or ability to discriminate differences in survival. For example, as shown in Fig 1, differences in survival are discriminated by patient groupings as defined within cell differentiation (T) or within lymph node metastasis (N). However, this analysis cannot distinguish the discriminative power of T variable (risk factor) from N variable (risk factor) (Fig 1) as these powers are not quantified. To compare discriminative powers between or among different risk factors so as to understand their powers in predicting survival prognosis, we have first used ROC curve and AUC to quantify the power of a survival predictor in gastric cancer, by which compares the potentials of different survival predictors (Fig 2 and Table 4). Similar AUCs suggest similar discriminative powers in predicting prognosis, which indicates the clinical usefulness of BMI, Alb, and TG in predicting survival among GC patients prior to surgery (Figs 3–5).

In summary, using a large sample of 320 GC patients with >10 years follow-up, our study demonstrates that preoperatively tested BMI, Alb, and TG are positively correlated with each other and they are protective factors for GC patients in terms of survival. We have first observed that BMI independently predicts survival for GC patients with a similar predicting power to postoperative TNM system. BMI, therefore, may be regarded as an indicator that reflects an overall physical status of patients integrating potential survival risk factors such as Alb and TG amongst many others yet to be revealed. In addition, we have first demonstrated that, in terms of survival analysis, predictor variables’ medians are useful in categorizing patient groups and, ROC curve and AUC area are a new method in quantifying predictive powers (abilities) for individual predictors in GC patients. As BMI, Alb and TG can be obtained before surgery and yet, they are first demonstrated to be negatively correlated with postoperative TNM staging system, these factors may serve as “pre-warning indicators” in management decisions before surgery. For example, when combined with other examinations such as clinical imaging, these “pre-warning indicators” may assist in personalized treatment/medicine for GC patients such as personalized surgical planning, optimal radio-chemotherapy and appropriate follow-up intervals after surgery. On the other hand, given the prognostic values of BMI, Alb, and TG for gastric cancer patients, it may be beneficial to adjust these parameters to normal or higher levels with appropriate treatments in addition to routine cancer therapies.

## Supporting Information

S1 Fig BMI is positively correlated with blood levels of triglyceride and albumin, respectively. Two typical correlation analyses using scatter diagram method are shown to illustrate positive correlations between BMI and triglyceride (left, *P*<0.05) and between BMI and albumin (right, *P*<0.01) which are 2 of the 66 paired correlation analyses presented in Table 2.
(TIF)

S2 Fig Kaplan-Meier survival analyses of BMI, Alb, and TG after removing 10 GC patients who died within 30 days after surgery. It is obvious that lower levels of BMI, Alb, and TG are correlated with poor overall survival among 310 GC patients after removing 10 GC patients who died within 30 days after surgery, in keeping with the observations shown in Figs 3, 4 and 5 with all 320 GC patients included. The observations shown here suggest that surgery- or other condition-related deaths may have minimal effect, if any, on survival in this GC patient population studied.
(TIF)
