# Supplementary figures and images for: Preoperative Body Mass Index, Blood Albumin and Triglycerides Predict Survival for Patients with Gastric Cancer
Source: PLoS One. 2016 Jun 16;11(6):e0157401. doi: 10.1371/journal.pone.0157401 (PMC4911005; doi:10.1371/journal.pone.0157401)

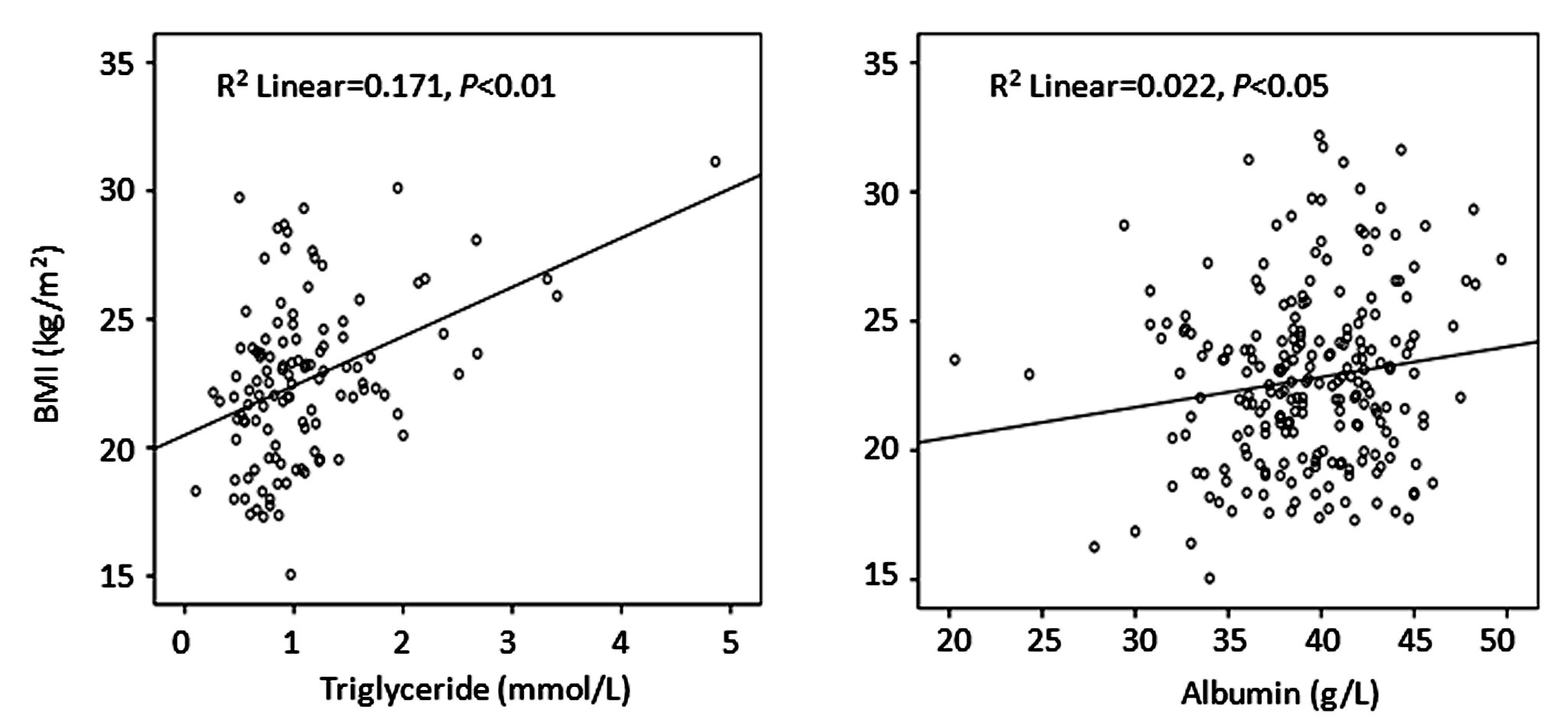

Supplement: S1 Fig — Two typical correlation analyses using scatter diagram method are shown to illustrate positive correlations between BMI and triglyceride (left, P<0.05) and between BMI and albumin (right, P<0.01) which are 2 of the 66 paired correlation analyses presented in Table 2. (TIF) [file pone.0157401.s001.tif]

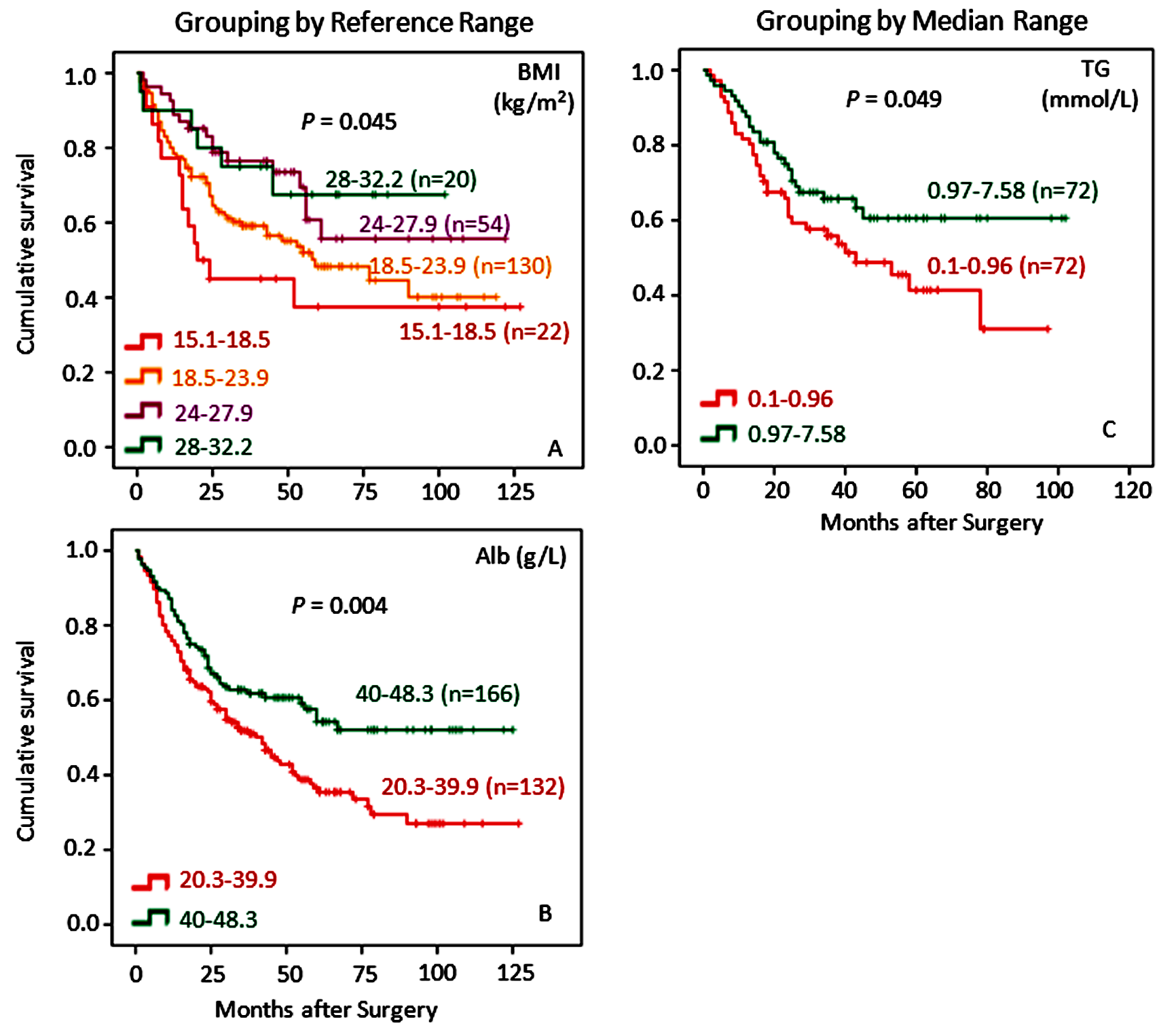

Supplement: S2 Fig — It is obvious that lower levels of BMI, Alb, and TG are correlated with poor overall survival among 310 GC patients after removing 10 GC patients who died within 30 days after surgery, in keeping with the observations shown in Figs 3, 4 and 5 with all 320 GC patients included. The observations shown here suggest that surgery- or other condition-related deaths may have minimal effect, if any, on survival in this GC patient population studied. (TIF) [file pone.0157401.s002.tif]
